# Dynamics of Human Endogenous Retroviruses Expression, Proviral Load and Systemic Inflammatory Status Modulated by Physical Exercise and Aging

**DOI:** 10.3390/ijms27073008

**Published:** 2026-03-26

**Authors:** Michelly Damasceno da Silva, Pablo Fortunato da Silva, Samuel Nascimento Santos, Matheus Esteves Fernandes, Maria Kauanne de Oliveira Santos, Camila Malta Romano, Jonatas Bussador do Amaral, Marina Tiemi Shio, Gislene Rocha Amirato, Carlos André Freitas dos Santos, Saulo Gil, André Luis Lacerda Bachi, Luiz Henrique da Silva Nali

**Affiliations:** 1 Post-Graduation Program in Health Sciences, Santo Amaro University, Rua Isabel Schimitt, 540, São Paulo 04743-030, Brazil; michelly.damasceno08@gmail.com (M.D.d.S.); pablofortunato1488@gmail.com (P.F.d.S.); samuel.santos3399@hotmail.com (S.N.S.);; 2 Hospital das Clinicas HCFMUSP, Faculdade de Medicina, Universidade de Sao Paulo, São Paulo 05508-070, Brazil; 3 Ent Research Laboratory, Department of Otorhinolaryngology-Head and Neck Surgery, Federal University of Sao Paulo, São Paulo 04039-020, Brazil; 4 Mane Garrincha Sport Education Center, Sports Department of the Municipality of São Paulo (SEME), São Paulo 04039-034, Brazil; 5 Discipline of Geriatrics and Gerontology, Department of Medicine, Paulista School of Medicine, Federal University of São Paulo, São Paulo 04039-020, Brazil

**Keywords:** HERVs, regular physical exercise, cytokines, inflammation, aging

## Abstract

Human endogenous retroviruses (HERVs), remnants of ancient germline infections, constitute ~8% of the human genome. Although mostly silenced, these elements can be expressed and play physiological or pathological roles. We investigated HERV expression dynamics, proviral load, and systemic inflammatory status in young and older adults, as well as the impact of regular physical exercise. PBMC and serum samples were collected from 30 young controls (YC), 30 inactive older adults (INAC) and 30 regularly exercising older adults (REG). Expression of HERV-W, -K, -H, Syncytin-1 and -2 was assessed by qPCR using the −2ΔΔCt method, and proviral load (HERV-W, -K, -H) was estimated by relative copy number. Serum cytokines (IL-1β, IL-6, IL-17, TNF-α, IFN-γ, IL-10) were quantified by ELISA. Statistical significance was set at *p* < 0.05. INAC participants showed higher proviral load of HERV-K, -W and -H compared to YC (*p* = 0.025), but overall lower HERV expression, except for HERV-K. REG presented increased expression of HERV-W (~1.5-fold, *p* < 0.0001), HERV-H (~1.8-fold, *p* < 0.0001; higher than YC *p* = 0.01), HERV-K (vs. YC *p* = 0.02) and Syncytin-1 (~1.4-fold vs. INAC and YC, *p* < 0.01). HERV-K was the most upregulated element in INAC. HERV-W and HERV-H expression were strongly correlated in all groups. INAC showed a pro-inflammatory profile, with elevated IL-6/IL-10, IL-1β/IL-10, and IFN-γ/IL-10 ratios. Older adults exhibit higher HERV proviral load, suggesting possible age-related insertions. Regular physical exercise modulates HERV expression, whereas inactivity is associated with reduced expression and increased inflammation. HERV-W and HERV-H maintain coordinated expression across ages, indicating interplay between inflammatory balance, aging, and retroviral activity.

## 1. Introduction

Human Endogenous Retroviruses (HERVs) are fossil viruses that have infected our ancestors’ germline cells millions of years ago [1,2]. They have passed through a complex process of interaction with their hosts that involved both horizontal and vertical transmission until fixation in the genome and perpetuation throughout the generations as Mendelian heritage [3]. Today, we know they compose around 8% of the human genome [4]. HERVs play several important roles in human physiology that vary from gene control regulation [5,6], a diverse and structured combined expression of many HERV families determines the pluripotency of cells and embryo development [7], to the placenta formation [8] and other fusogenic activities such as the formation of osteoclast cells [9]. In the other hand, the abnormal expression of HERVs may be associated with autoimmune diseases, such as Multiple Sclerosis, which varies from higher level of expression [10,11,12,13,14], protein detection in brain lesions [11,15], immunopathological response through TLR-4 evidence [16] and induction of experimental model MS in mice exposed to HERV-W-env protein [17], in fact this disease gathers the highest number of evidences. However, data also point to the role of HERVs in Rheumatoid Arthritis [18], Systemic Erythematosus Lupus [19] and type 1 diabetes [20] pathogenesis. Also, HERVs seem to be associated with other aging-associated diseases such as cancer [21,22,23].

Beyond these aspects, it has been reported that HERV expression dynamics may be impacted by several conditions, particularly inflammation response [24] and interaction with other infectious agents [25,26,27,28,29]. Another factor that could be relevant for inducing the expression of HERVs is related to the practice of physical exercise. In this respect, two previous studies have described that HERVs may be upregulated in: (i) long-term endurance athletes, since they exhibit high levels of expression of many HERV families, including the Syncytin-1 post-competitive season [30]; and (ii) acutely after a single resistance training in regularly exercising individuals [31]. It is of overriding importance to describe that HERVs’ expression is a key factor in myogenesis and may interact with caveolins [32]. This striking piece of evidence points to the role of these retroelements as potential allies in the physiological muscle repair process.

Added to this complex behavior of these domesticated retroviruses’ genes, it is well known that some aging-associated phenomena, such as inflammaging, which translates to a chronic systemic, sterile pro-inflammatory status associated with aging, may significantly impact healthy aging [33], contributing to the senescence and increasing the manifestation of diseases related to aging [34]. However, it has been highlighted that regular exercise training can mitigate both the development and progression of inflammaging [35,36]. It is worth mentioning that, to date, there is a lack of data describing the dynamics of HERV’s expression throughout aging and how regular physical exercise impacts its expression. Therefore, in the present study, we present data regarding not only the proviral load of HERVs, but also the dynamics of distinct HERV families’ expression in young adults and older adults. Additionally, we investigated the impact of regular physical exercise on HERV’s expression, and finally, assessed the association of these elements with the systemic inflammatory status of these volunteer groups.

## 2. Results

As described, a total of 90 individuals were enrolled in the study. In Table 1, the sociodemographic information of the individuals is described.

### 2.1. HERVs Proviral Load in Young and Older Adults

**Figure 1:**
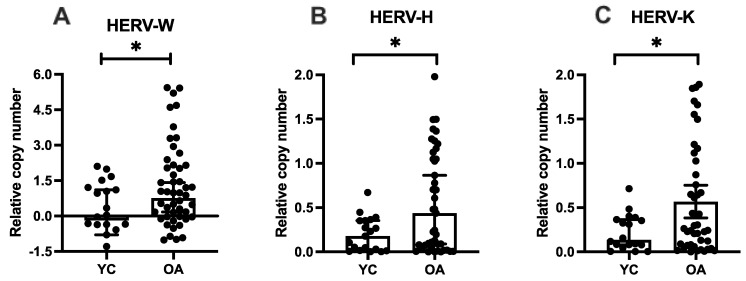
Proviral load of HERVs in young and older adults in relative copy number to GAPDH endogenous control. (**A**) HERV-W-env * *p* = 0.03, (**B**) HERV-H-pol * *p* = 0.05, (**C**) HERV-K-gag * *p* = 0.02. Legends: YC = Young Controls, OA = Older Adults. Mann–Whitney test was used, bars show median and lines 95% Confidence Interval.

Relative copy number of HERVs revealed that older adults exhibit higher proviral load of HERV-W (*p* = 0.03), HERV-H (*p* = 0.05) and HERV-K (*p* = 0.02) than young adults (Figure 1).

**Figure 2:**
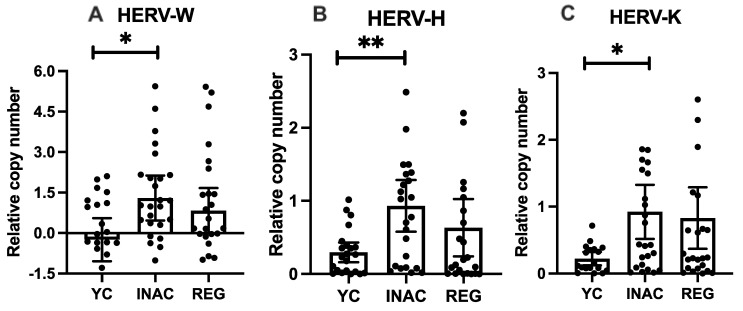
Proviral load of HERVs in young, inactive older adults and regularly exercised older adults in relative copy number to GAPDH endogenous control. (**A**) HERV-W-env * *p* = 0.04, (**B**) HERV-H-pol ** *p* = 0.01, (**C**) HERV-K-gag * *p* = 0.03. Legends: YC = Young Controls, INAC = Inactive Older Adults Group, REG = Regularly Exercised Older Adults. Mann–Whitney test was used, bars show median and lines 95% Confidence Interval.

Next, we evaluated the proviral load of each HERV family across the groups. The data showed that INAC individuals exhibited significantly higher relative copy number of HERV-W (*p* = 0.04), HERV-H (*p* = 0.01), and HERV-K (*p* = 0.03) compared with the young group. In contrast, although the REG appeared to display a higher copy number of HERVs, no statistically significant differences were observed. Likewise, no differences were observed between the older adults’ groups. These results can be seen in Figure 2.

### 2.2. HERVs Expression in Young Individuals, Regularly Exercised and Inactive Older Adults

**Figure 3:**
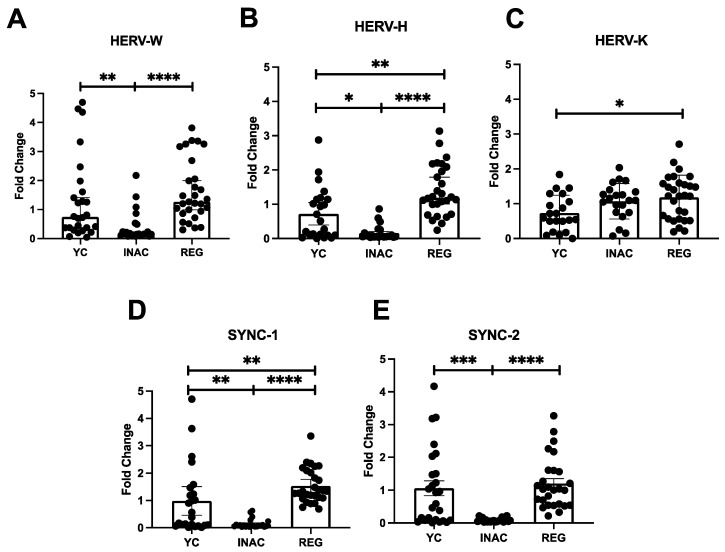
The Level of HERV expression in young, inactive older adults and regularly exercised older adults expressed in fold change (2^−ΔΔCt^). (**A**) HERV-W-env, (**B**) HERV-H-pol, (**C**) HERV-K-gag, (**D**) SYncytin-1 and (**E**) Syncytin-2 Legends YC = Young Controls Group, INAC = Inactive Older Adults Group, REG = Regularly Exercised Older Adults Group, * = *p* < 0.05, ** = *p* < 0.01, *** = *p* < 0.001, **** = *p* < 0.0001. Mann–Whitney test was used, bars show median and lines 95% Confidence Interval.

All individuals presented detectable levels of all HERVs as well as Syncyntin-1 and 2. In addition, the groups exhibited distinct expression profiles across the different HERV families. Notably, HERV-W (Figure 3A), HERV-H (Figure 3B), Syncytin-1 (Figure 3D) and Syncytin-2 (Figure 3E) were downregulated (fold change < 1.0) in INAC individuals when compared to YC and REG. This downregulation was statistically significant when comparing INAC to YC and REG for HERV-W (*p* < 0.01 and *p* < 0.0001), HERV-H (*p* = 0.01 and *p* < 0.0001), Syncytin-1 (*p* < 0.01 and *p* < 0.0001), and Syncytin-2 (*p* < 0.001 and *p* < 0.0001). In contrast, YC individuals presented low expression levels for all HERVs, while REG individuals presented low expression of HERV-W (Figure 3A) and Syncytin-2 (Figure 3E). Interestingly, although not exhibiting markedly elevated expression, REG individuals showed increased levels of HERV-H (~1.3-fold change, *p* < 0.01) and Syncytin-1 (1.5-fold change, *p* < 0.01) expression compared to YC. Distinct from the other families, HERV-K expression was not downregulated in INAC individuals (Figure 3C), and REG individuals showed higher HERV-K expression than YC (*p* < 0.05). All findings are summarized in Figure 3.

**Figure 4:**
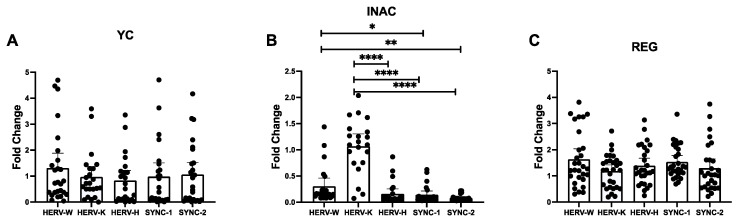
Dynamics of different HERV families’ expression (HERV-W-env, HERV-K-gag, HERV-H-pol) and Syncytin-1 and 2 in the different groups. (**A**) YC, (**B**) INAC, (**C**) REG. Data are expressed in fold change (2^−ΔΔCt^). Legends YC = Young Controls Group, INAC = Inactive Older Adults Group, REG = Regularly Exercised Older Adults Group, * = *p* < 0.05, ** = *p* < 0.01, **** = *p* < 0.0001. Mann–Whitney test was used, bars show median and lines 95% Confidence Interval.

In Figure 4, we summarize the analysis of the dynamics of the HERV family’s expression level according to each group individually. The analysis revealed an imbalance of HERV expression in INAC individuals. HERV-K (Figure 4B) was upregulated when compared to HERV-H *p* < 0.0001), Syncytin-1 (*p* < 0.0001) and Syncytin-2 (*p* < 0.0001), and HERV-W, which is downregulated in INAC individuals, was slightly higher than Syncytin-1 (*p* < 0.05) and Syncytin-2 (*p* < 0.01). No difference was observed regarding the level of specific HERV family in YC (Figure 4A) and REG (Figure 4C).

**Figure 5:**
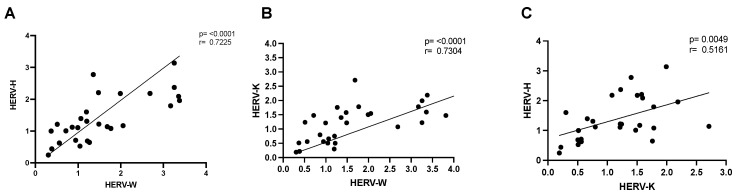
Spearman’s correlation coefficient analysis indicates a positive correlation between the level of HERV expression in REG individuals. (**A**) HERV-H vs. HERV-W, (**B**) HERV-K vs. HERV-W and (**C**) HERV-H vs. HERV-K. Data are expressed in fold change.

Finally, we have assessed the possible correlation of the HERV family’s expression between the differentially upregulated HERVs. The Spearman correlation coefficient analysis revealed that REG individuals presented positive correlation between HERV-H and HERV-W (r = 0.722, *p* < 0.0001), HERV-H and HERV-K (r = 0.516, *p* < 0.01), and HERV-K and HERV-W (r = 0.730, *p* < 0.0001). These findings can be observed in Figure 5. All the other correlation analyses are available in Supplementary Table S1.

### 2.3. Systemic Inflammatory Status Analysis

**Figure 6:**
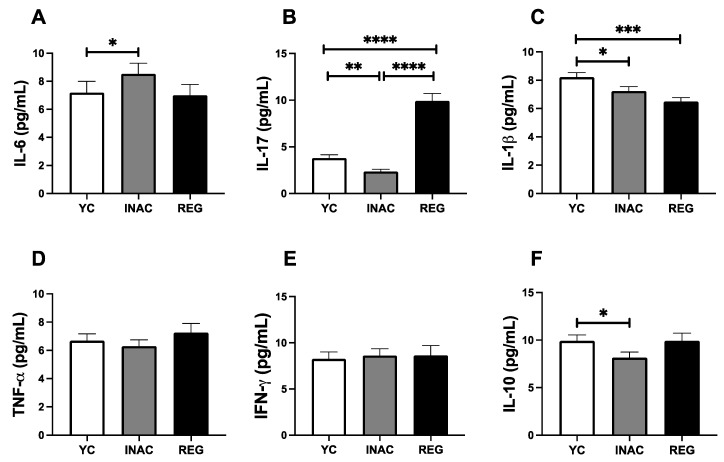
Serum concentration of cytokines in young, inactive older adults and regularly exercised older adults. IL-6 (**A**), IL-17 (**B**), IL-1β (**C**), TNF-α (**D**), IFN-γ (**E**) and IL-10 (**F**) Legends YC = Young Controls Group, INAC = Inactive Older Adults Group, REG = Regularly Exercised Older Adults Group, * = *p* < 0.05, ** = *p* < 0.01, *** = *p* < 0.001, **** = *p* < 0.0001. Mann–Whitney test was used, bars show median and lines 95% Confidence Interval.

To further characterize the systemic inflammatory status across the groups, Figure 6 describes the circulating concentration of cytokines. We observed that INAC individuals presented higher IL-6 concentrations compared with YC (*p* < 0.05, Figure 6A). REG present higher IL-17 levels relative to both YC and INAC (*p* < 0.0001 for both comparisons; Figure 6B), while YC displayed higher concentration than INAC (*p* < 0.01). YC individuals also demonstrated higher IL-1β levels compared with INAC and REG (*p* < 0.05 and *p* < 0.001, respectively, Figure 6C). Finally, INAC individuals present a lower concentration of IL-10 than YC (*p* < 0.05, Figure 6F).

**Figure 7:**
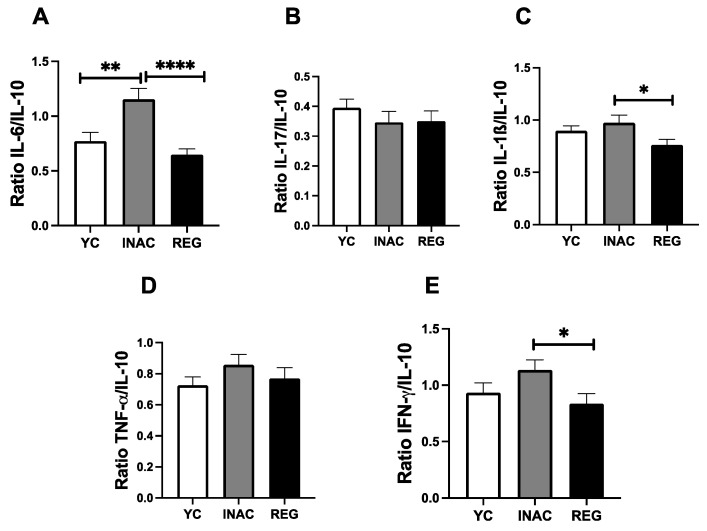
Pro-inflammatory and anti-inflammatory cytokine ratio concentration in serum of the volunteers of the study in young, inactive older adults and regularly exercised older adults. Ratio IL-6/IL-10 (**A**), Ratio IL-17/IL-10 (**B**), Ratio IL-1 β/IL-10 (**C**), Ratio TNF-α/IL-10 (**D**) and Ratio INF-γ/IL-10 (**E**) Legends YC = Young Controls Group, INAC = Inactive Older Adults Group, REG = Regularly Exercised Older Adults Group, * = *p* < 0.05, ** = *p* < 0.01, **** = *p* < 0.0001. Mann–Whitney test was used, bars show median and lines 95% Confidence Interval.

**Figure 8:**
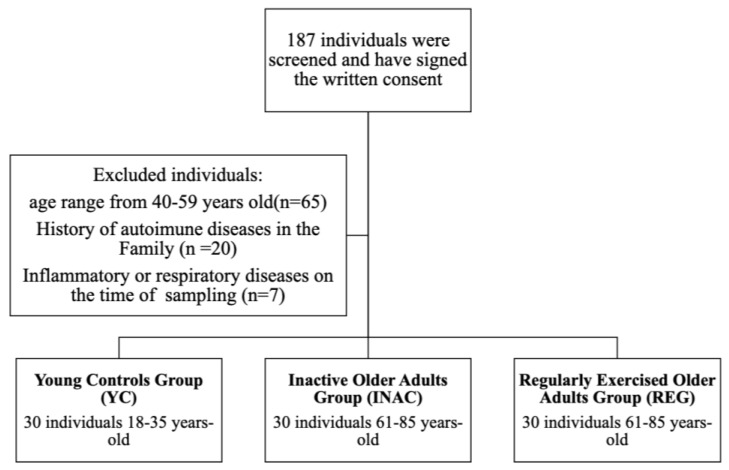
Flowchart of the volunteer recruitment process for the research.

Next, we sought to determine the ratio between the pro-inflammatory cytokines and the anti-inflammatory cytokine IL-10. We have observed that INAC individuals presented a marked pro-inflammatory profile when compared to other groups. In sum, we observed that INAC showed a higher ratio of IL-6/IL-10 than YC and REG (*p* < 0.01, *p* < 0.0001 Figure 7A), as well as IL-1β/IL-10 (*p* < 0.05 Figure 7C) and IFN-γ/IL-10 (*p* < 0.05 Figure 7E) than REG individuals. All these findings can be observed in Figure 8.

## 3. Discussion

Based on the results, we have observed intriguing findings regarding the patterns involving HERVs and the human genome in two distinct periods of life. The first finding describes the significant increase in the proviral load of HERV-W-env, HERV-H-pol and HERV-K-gag in older adults. This finding might reveal a groundbreaking scenario, where HERVs may spread within the genome by retrotransposition [37], and this may result in an increase in the total amount of HERV elements within the human genome throughout the aging of individuals. Also, this seems to be observed systematically and to be a behavior presented by different HERV families. This is interesting, since the distinct family of HERVs has passed through the endogenization process in different periods of evolution [7], and still they seem to preserve their function and be able to retrotranspose in the genome, leading to an increase in the proviral load. Importantly, our findings are in line with a previous comprehensive study that has described that aged cells present higher HERV-K proviral components compared to non-aged/senescent cells [38]. The authors also emphasize that HERV-K expression is upregulated and may represent an impact on the epigenetics of these cells, possibly driving cell senescence [38]. Here, we describe that this increased proviral load may also occur with other HERV families, which suggests that other HERVs might also be implicated in aging. Interestingly, this result reflects a potential and complex interaction that possibly can drive the expression of HERVs throughout life to be regulated by many conditions. Among such conditions, we highlight that HERV expression can be impacted by the inflammation [3,39,40,41,42], chronic oxidative stress [43,44,45] and interaction with infectious agents, especially other viruses [26,28,46,47,48,49]. In this sense, it is feasible to address that the human organism is exposed to many factors that lead to HERV expression, and then its activation and retrotransposition are likely to occur many times throughout life. Importantly, the specific consequences of this increase in the proviral load for aging issues, associated with the occurrence of certain diseases and also the potential of these de novo retroinsertions to be transmitted to further generations through the gamete cells, remain to be elucidated and should be addressed in further investigations. Importantly, it is not yet clear how these possible retro insertions may impact aging-associated diseases, and follow-up studies and whole genome sequencing in order to identify these retro insertion locations should also be addressed in further studies.

On the other hand, we have also described findings of the dynamics of HERV expression in young adults, inactive older adults, and regularly exercised older adults. As described, the HERV proviral load was similar between older adults regardless of whether they were practitioners of physical exercise or not. However, the level of expression was distinct between the groups. Except for HERV-K, all HERVs were downregulated in the INAC individuals, and REG individuals presented low, and for some HERVs, exhibited a slight increase in the fold change in HERV expression. These findings appear to indicate that regular physical exercise might directly or indirectly impact the expression of HERVs. Yet for unknown reasons, sedentarism can induce a downregulation of transcriptional HERV activity, reflecting a reduction in the functional demand of these elements in a physiological context of reduced metabolic and regenerative stimulation. Conversely, we found that REG presents increased levels of HERV-H and Syncitin-1 expression when compared to YC. In fact, these findings were also observed in different contexts of physical exercise such as Long-term endurance athletes that presented higher level of HERVs expression, including Syncytin-1, post-competitive season [30], and also in a single acute strength exercise session modulates positively the levels of HERVs expression in regularly exercise young adults [31], which reinforce the idea that physical exercise might be a key factor in modulating the HERVs expression.

While Syncytin-1 exhibits an important fusogenic role and plays a key role in myogenesis [32], HERV-H is associated with gene regulation and pluripotency [7]. HERV-H, along with other elements, has acted as a regulator of the immune system [50,51]. Altogether, these HERVs may respond in consonance to the regular physical exercise since in REG HERVs were positively correlated, and the increase in Syncytin-1 reinforces the proposal that regular physical exercise may promote the transcriptional, and maybe beneficial, activity of HERVs.

It is remarkably important to emphasize that the increased level of expression of some HERVs in REG was way lower than previous reports that showed higher fold change in some diseases when compared to the expression level described here [10,11,14,52,53]. In this sense, the slightly higher increase in the HERV expression, especially HERV-W/Syncytin-1 and HERV-H, seems to present a physiological key factor found in older individuals who are regularly exercising.

HERV-K was upregulated in INAC individuals when compared to other HERVs. This was the only HERV family not downregulated in this group. Although HERV-K is not usually associated with autoimmune diseases, it has been associated with some diseases such as cancer [54,55], ALS [56,57] and schizophrenia [58]. Also, HERV-K seems to be involved in the cell senescence process, since it was evidenced that senescent cells accumulate HERV-K proteins and trigger an innate inflammatory response. Added to this finding, our results point to the fact that HERV-K may be used as a biomarker for senescence and aging and should be further investigated in order to better understand its involvement in aging and with other senescence-associated diseases.

Beyond these findings, another interesting result verified here was that INAC presented a higher systemic concentration of some pro-inflammatory cytokines, especially IL-6, and, particularly with the reduction in IL-10, a prominent systemic pro-inflammatory status when compared to YC and REG. Taken together, these observations can demonstrate that not only is the inflammaging phenomenon present in those volunteers, but also corroborate the literature that claims that long-standing regular exercise practice is able to elicit a balanced systemic inflammatory status [36,59], including the older population [36,60,61]. Of note, gradual and chronic elevations of circulating IL-6 levels have been reported during aging [62], which leads to this cytokine being a pillar of inflammaging [33,63]—a key factor to induce cell senescence [34,64] and age-related diseases [65]. Although the isolated analysis of IL-6 allows us to suggest this scenario, the higher IL-6/IL-10 ratio in INAC than the other groups reinforces our proposition that the manifestation of the inflammaging phenomenon is closely associated with a lifestyle without regular practice of physical exercise. At this point, it is important to mention that the ratio analysis between pro- and anti-inflammatory cytokines has been pointed out as an accurate way to identify the balance/imbalance of the inflammatory process, which includes the older population [66,67]. Furthermore, the higher IL-1β/IL-10 and IFN-γ/IL-10 ratios in INAC than REG corroborate our suggestion that the long-standing regular practice of physical exercise positively impacts systemic inflammatory status in the older adult population. In the HERV context, in an interesting way, it was reported that HERV-K may impact innate immune response through the GAS-Stimulator of interferon genes (STING) pathway [38]. Based on it, we could putatively suppose that the increase in HERV-K expression may impact the systemic elevation of IL-6, or even in the IL-6/IL-10 and IFN-γ/IL-10 ratios, particularly in INAC, thus demonstrating a plausible role of HERV-K expression in inflammaging. Besides these data, it is of utmost importance to mention that even though the higher circulating IL-17 levels in REG could indicate that a pro-inflammatory status is present, according to the literature, long-standing exercise training can lead to an increase in systemic IL-17 levels [68], which can be associated with an improvement of the immune response, since it was demonstrated that a Th17 profile, in which IL-17 is one of the main players, helps the immunity to respiratory virus vaccination [69,70,71]. Thus, the increased IL-17 levels in REG as compared to INAC could indicate that a proper immune response was maintained in those older adults. Lastly, the observation of reduced IL-1β levels in the older adult groups, regardless of the exercise training, even though it can be different from expected, it is worth mentioning that it was previously reported that their levels in healthy young people were not different from those in older adults [72]. Based on this finding, what we can suggest is that the long-standing regular exercise practice is able to benefit the older adults by reducing the circulating levels of this pro-inflammatory cytokine, thus, consequently, mitigating the inflammaging.

Our study presents some limitations that should be addressed: (i) our study population did not include middle-aged individuals, which could aid in describing the accumulation of the proviral load in distinct moments of life, and (ii) all INAC individuals had comorbidities, and it must be taken into account that this likely has some impact on HERV expression. However, our findings may elucidate the condition itself and maybe HERV expression and proviral load might be consequence of these conditions as well; (iii) although the primers are specific for each HERV gene, it is not possible to determine the origin of these expression since HERVs may retrotranspose and/or some loci might be silenced or able to be transcribed, and; (iv) proviral load was performed by relative quantification based on a housekeeping gene, although not absolute quantification was not performed, we might rely on a variation in assays efficiencies, which might have impacted in the study. Also, we have performed a cross-sectional study, and we did not follow up these individuals in order to observe the retroinsertion of HERVs in real time. However, we may assume that possible assay variation would be the same in all studied groups, which could bring evidence of real variation in proviral load through age, and although our shows that older individuals present higher HERVs proviral load and retroinsertion is our main hypothesis, other possible mechanisms should not be excluded, such as PBMC composition changes between the age individuals or clonal expansion and maybe the possible exercise role impact in these cells composition and should this be investigated further.

## 4. Materials and Methods

### 4.1. Study Population and Ethical Considerations

This is a cross-sectional case-control study.

Individuals were selected according to Figure 8, using the following criteria:

Young Controls Group (YC): Individuals ranging from 18 to 35 years old who present healthy conditions. These individuals were recruited at the Universidade Santo Amaro.

Inactive Older Adults Group (INAC): Individuals ranging from 61 to 85 years old. These individuals were recruited during their registration on the Sports and Physical Activity Program from Universidade Santo Amaro (PAEC), which is an extension program focused on promoting physical activity to people of all ages. Sampling occurred before the start of the physical exercise program. It was mandatory that the individuals not perform any physical exercise protocol prior to the registration.

Regularly Exercised Older Adults Group (REG): Individuals ranging from 61 to 85 years old. These individuals were regular exercise practitioners, and they were recruited to the Exercise Program for Longevity of the Ibirapuera Olympic Center at the “Mané Garrincha” Sports and Education Center in São Paulo, Brazil.

For all groups, patients who presented autoimmune diseases, or familial history of autoimmune diseases, who were under treatment with corticosteroids, under treatment for cancer and presented respiratory tract symptoms at the moment of the sampling were not included in the study.

Sociodemographic information, clinical findings and regular use of medications were obtained. Importantly, for this study, we have followed the Strengthening the Reporting of Observational Studies in Epidemiology (STROBE) guideline for case-control studies [73]. This study was approved by the ethical committee of Universidade Santo Amaro under protocol #6.254.440. All volunteers were included in the study after signing a written consent, which was also approved by the ethical committee. This study was carried out in agreement with the Declaration of Helsinki and ethical guidelines outlined in Resolutions 466/2012 and 510/2016 of the Brazilian National Health Council.

### 4.2. Physical Exercise Protocol for REG Individuals

The individuals included in this group were performing the regular training protocol for at least 3 years without interruptions.

REG participants followed a structured training protocol comprising (i): aerobic exercise performed at 60–70% of the heart rate reserve, with maximal heart rate estimated by the Tanaka equation (208 − 0.7 × age) [74], involving low-impact step-platform activities, jumps, coordination drills, and rhythmic movements (occasionally including dance), with monthly cardiac monitoring using a Polar FT1 device; and (ii): resistance training consisting of at least five exercises targeting distinct muscle groups (upper and lower limbs, abdomen, gluteal muscles, and core/postural stabilizers, including dorsal and lumbar muscles), executed slowly in two sets of 10–20 repetitions at 50–60% of one-repetition maximum, arranged in four consecutive sessions combining two muscle groups per session, with monthly load adjustments guided by the Borg scale.

### 4.3. Blood Samples Collection and Preparation

Blood samples were collected in two tubes: 1 EDTA tube to obtain PBMC for molecular analysis, and 1 gel-barrier dry tube to obtain serum aliquots, which were used in cytokine concentration analysis after centrifugation and collection of the serum. PBMCs were obtained by the Ficoll-Hypaque (GE Healthcare, Chicago, IL, USA) protocol, and nucleic acid was obtained through the Trizol/chloroform protocol. Briefly, Ficol-Hypaque was added to whole blood in a 1:1 proportion and centrifuged at 800× *g* for 20′, and the upper solution (containing PBMC) was collected. Then the solution was washed repeatedly with sterile PBS and centrifuged until complete removal of the residual erythrocytes. The nucleic acid was obtained from the dry cell pellet samples with the following protocol: 1 mL of Trizol was added to each of the cell pellet samples, and up-down was performed until complete homogenization, then 200 µL of chloroform was added, and samples were centrifuged at 10,000× *g* at −4 °C. The upper phase was completely removed (around 600 µL), and then nucleic acid was precipitated with Isopropanol 100% and washed twice with 70% Ethanol. Nucleic acid was resuspended in 40 µL nuclease-free H_2_O. An aliquot of 10 µL was used to perform the proviral load protocol, which will be further explained in 4.5. The heading and the leftover sample were submitted to a rigorous protocol to remove the genomic DNA, which was performed with DNA-free Turbo (Ambion, Berlin, Germany). Absence of contaminant genomic DNA was confirmed by Real Time PCR with primers complementary to the GAPDH gene, in the absence of Reverse Transcriptase. RNA was quantified and then stored at −80 °C. Around 150 ng of RNA was used to perform the cDNA synthesis with the High-Capacity cDNA Reverse Transcription Kit (ThermoFisher, Waltham, MA, USA).

### 4.4. HERV Detection and Relative Quantification Analysis

HERV expression was qualitatively assessed, in terms of presence and absence of expression, and relatively quantified in all samples. Real-time reverse transcription PCR (qRT-PCR) for HERV-W-env, HERV-K-gag and HERV-H-pol, Syncytin-1 and 2 were performed, using the primers described in Table 2.

The qRT PCR mix included 0.1 μM of each primer, 1x of Power-up PCR Master Sybr Green (Thermofisher, Waltham, MA, USA), 3 μL of the normalized cDNA preparation and nuclease-free H_2_O in a final mix volume of 25 μL. For all HERVs, the cycling conditions were as follows: 95 °C for 10 min followed by 40 cycles of 95 °C for 1 min, 50 °C for 45 s and 60 °C for 1 min. For the GAPDH, the assay cycling conditions were: 37 °C for 30 min for the cDNA construct step, 95 °C for 10 min, followed by 40 cycles of 95 °C for 1 min and 60 °C for 1 min. A final cycle to determine the melting temperature of the testing samples (50 °C to 95 °C).

The level of expression was determined by calculation of 2^−ΔΔCt^, where ΔCt = (INAC or REG HERV Ct − INAC or REG GAPDH Endogenous Control Ct) − (Average of ΔCt of all controls), and the results were represented in fold change. Positive samples were confirmed by standard melting curve analysis. Genomic DNA was used as a positive control, and samples (PCR mix and H_2_O) in the absence of DNA were used as negative controls; they were added in every reaction. All reactions were performed in the StepOne Plus Real-Time PCR.

### 4.5. Proviral Load Quantification of HERV-W, HERV-K and HERV-H

To determine the proviral load, genomic DNA was extracted from the dry cell pellet using the PureLink Genomic DNA Mini Kit (Thermofisher). Total DNA was quantified and normalized to 400 ng per reaction. The total amount of HERV copies per cell was estimated relative to the stable control gene GAPDH, which contains two copies per cell [81,82]. Importantly, all PCR assays present similar efficiency, assuming minimal variation between the assays. Similarly, to the relative quantification analysis, we have performed ΔΔCt analysis *2 considering the diploid profile of the GAPDH gene.

### 4.6. Determination of Systemic Cytokine Concentration

Serum concentrations of the cytokines IL-1β, IL-6, IL-10, IL-17, TNF-α, and IFN-γ were determined using commercial ELISA kits (ThermoFisher) following the manufacturer’s instructions. Cytokine concentrations were calculated from standard curves with correlation coefficients ranging from 0.95 to 0.99. Intra-assay coefficients of variation ranged from 2.5% to 4%, and inter-assay coefficients of variation ranged from 8% to 10%.

### 4.7. Sample Size Calculation and Statistical Analysis

Sample size was initially considered by previous studies of our group [31,52,53,83]. Given that previous studies show that most individuals exhibit low levels of HERV expression, detecting at least one individual expressing HERVs is highly likely, with minimal risk of error. Nevertheless, to ensure an effect size of 80%, we performed a sample size calculation using the Z-approximation test for means, which indicated that 27 participants per group would be required to achieve a 95% confidence level. The significance analysis of the quantitative variables was initially analyzed regarding the normal distribution of the data with the Shapiro–Wilk test. Since the data were not normally distributed, the non-parametric test Mann–Whitney was used to compare the median scores between two independent groups, Kruskall-Wallis for intergroups analysis and Spearman coefficient test for correlation analysis. All tests were conducted under the assumption of a first-type error probability (alpha) of 5%.

## 5. Conclusions

Our findings highlight the dynamics of HERV expression and proviral load in young adults, inactive older adults, and regularly exercising older adults, providing insights into how these elements behave during aging. We observed an age-associated increase in HERV proviral load, suggesting that elevated HERV copy numbers may represent a characteristic feature of the aging process. Furthermore, our results reinforce the potential modulatory role of physical exercise on HERV expression. The observed patterns indicate that HERV activity might contribute to tissue repair, maintenance of physiological homeostasis, and possibly to the regulation of inflammatory responses in older adults. Collectively, these data add information that HERV elements might be associated with aging-associated processes and highlight HERV-K as a potential biomarker of aging and possibly cellular senescence.

## Figures and Tables

**Table 1:** Sociodemographic and clinical information of the participants. Legend: * shows the statistically different comparisons between specific groups. YC = Young Controls Group, INAC = Inactive Older Adults Group, REG = Regularly Exercised Older Adults Group (REG), Avg = Average, SD = Standard Deviation, F = Female, M = Male, NS = Not significant, BMI = Body Mass Index.

Variable	YC (18–35 Years-Old)	INAC (≥60 Years-Old)	REG (≥60 Years-Old)	*p*
N	30	30	30	
Age (avg ± SD)	29 ± 4 *	69 ± 6	71 ± 5	* 0.0001
Sex (F/M)	21/9 (70/30%)	18/12 (60/40%)	22/8 (73/27%)	NS
Ethnic (%)	Asian: 1 (3)	White: 13 (43)	Asian: 13 (43)	
	White: 19 (63)	Black: 7 (23)	White: 8 (27)	NS
	Black: 5 (17)	Brown: 10 (34)	Black: 5 (17)	
	Brown: 5 (17)		Brown: 4 (13)	
BMI (kg/m^2^, avg ± SD)	25.0 ± 4.1	29.4 ± 4.8	24.7 ± 4.3	INAC vs. REG 0.0001 *YC vs. INAC* *0.0001*
Comorbidities (n)	none	Prediabetes (7) Diabetes (10) Systemic Arterial Hypertension (15) Osteoarthritis (3) Sarcopenia (8) Myocardial infarction (3) Hypercholesterolemia (10)	Prediabetes (5) Systemic Arterial Hypertension (6) Sarcopenia (1) Hypothyroidism (1) Hypercholesterolemia (1)	NS
Regular use of medications	No *	Yes (all of them)	Yes 8/27% No 22/73%	* *INAC vs. YC 0.0001*
Medications (n)		Hydrochlorothiazide (8) Enalapril (10) Insulin (9) Glucophage (5) Simvastatin (11) Losartan (5) Metformin (7) Doxazosin (2) Thiamine (2) Sarcoplex (7) Acetylsalicylic acid (6)	Levothyroxine (1) Hydrochlorothiazide (1) Rosuvastatin (2) Glucophage (4) Ezetimibe (1) Amlodipine (1)	

**Table 2:** Sets of primers used on the Real Time PCR assays to detect HERV expression and to determine the proviral load.

Oligonucleotides	Forward Primer (5′-3′)	Reverse Primer (5′-3′)
HERV-W env [75]	CCAATGCATCAGGTGGGTAAC	GAGGTACCACAGACAAAAAATATTCCT
HERV-H pol [76]	CACGTTTTATCCGTGGACCC	AGGCATCCCTGCAATGATTAA
HERV-K gag [77]	AGCAGGTCAGGTGCCTGTAACATT	TGGTGCCGTAGGATTAAGTCTCCT
Syncytin-1 [78]	ATGGAGCCCAAGATGCAG	AGATCGTGGGCTAGCA
Syncytin-2 [79]	TTGGGTAAATCAATCAGGAAAAGT	GTAAACTGGAGGCTTGATTTAGGA
GAPDH [80]	ACCCACTCCTCCACCTTTGAC	TGTTGCTGTAGCCAAATTCGTT

## Data Availability

In accordance with ethical and confidentiality requirements, the datasets supporting the findings of this study are not publicly available but can be obtained from the corresponding authors upon request.

## Supplementary Materials

The following supporting information can be downloaded at: https://www.mdpi.com/article/10.3390/ijms27073008/s1.

## Abbreviations

The following abbreviations are used in this manuscript:

ALS	Amyotrophic Lateral Sclerosis
BMI	Body Mass Index
cDNA	Complementary DNA
Ct	Cycle Threshold
DNA	Deoxyribonucleic Acid
EDTA	Ethylenediaminetetraacetic Acid
ELISA	Enzyme-Linked Immunosorbent Assay
F/M	Female/Male
GAPDH	Glyceraldehyde-3-Phosphate Dehydrogenase
H_2_O	Water
HERV	Human Endogenous Retrovirus
HERV-H	Human Endogenous Retrovirus Family H
HERV-K	Human Endogenous Retrovirus Family K
HERV-W	Human Endogenous Retrovirus Family W
IFN-γ	Interferon Gamma
IL	Interleukin
INAC	Inactive Older Adults Group
OA	Older Adults
PBMC	Peripheral Blood Mononuclear Cells
PBS	Phosphate-Buffered Saline
PCR	Polymerase Chain Reaction
REG	Regularly Exercised Older Adults Group
RNA	Ribonucleic Acid
RT-PCR	Reverse Transcription Polymerase Chain Reaction
SD	Standard Deviation
STING	Stimulator of Interferon Genes
STROBE	Strengthening the Reporting of Observational Studies in Epidemiology
SYBR	SYBR Green Dye
TNF-α	Tumor Necrosis Factor Alpha
YC	Young Controls Group
