# Supplementary material for: Dynamics of Human Endogenous Retroviruses Expression, Proviral Load and Systemic Inflammatory Status Modulated by Physical Exercise and Aging
Source: Int J Mol Sci. 2026 Mar 26;27(7):3008. doi: 10.3390/ijms27073008 (PMC13073308; doi:10.3390/ijms27073008)
Supplement: Supplementary file 1 [file ijms-27-03008-s001.zip › ijms-4198172-supplementary.pdf]

Table S1. Spearmann's correlation analysis between HERVs and cytokines in all groups. Legends YC= Young Controls Group, INAC= Inactive Older Adults Group, REG= Regularly Exercised Older Adults Group, * indicates significant finding.

Group	HERV	Cytokine	Spearman_r	p_value	Significant(*)
YC	HERV-K	IL6	-0,2529	0,2443	N/S
INAC	HERV-K	IL6	-0,2782	0,2217	N/S
REG	HERV-K	IL6	0,2543	0,8952	N/S
YC	HERV-K	IL17	-0,0816	0,7149	N/S
INAC	HERV-K	IL17	0,0716	0,7279	N/S
REG	HERV-K	IL17	-0,3160	0,0496	*
YC	HERV-K	IL1β	-0,1430	0,5113	N/S
INAC	HERV-K	IL1β	-0,1216	0,6149	N/S
REG	HERV-K	IL1β	-0,1116	0,5349	N/S
YC	HERV-K	IL10	-0,0186	0,7230	N/S
INAC	HERV-K	IL10	0,0652	0,7849	N/S
REG	HERV-K	IL10	0,4178	0,3756	N/S
YC	HERV-W	IL6	0,1230	0,2630	N/S
INAC	HERV-W	IL6	-0,1269	0,9460	N/S
REG	HERV-W	IL6	0,0365	0,8450	N/S
YC	HERV-W	IL17	-0,3980	0,2633	N/S
INAC	HERV-W	IL17	0,3120	0,1997	N/S
REG	HERV-W	IL17	0,2310	0,5156	N/S
YC	HERV-W	IL1β	-0,1650	0,3920	N/S
INAC	HERV-W	IL1β	-0,4325	0,0309	*
REG	HERV-W	IL1β	0,1260	0,9493	N/S
YC	HERV-W	IL10	-0,7522	0,7149	N/S
INAC	HERV-W	IL10	0,3202	0,8867	N/S
REG	HERV-W	IL10	-0,0623	0,0749	N/S
YC	HERV-H	IL6	-0,1652	0,4577	N/S
INAC	HERV-H	IL6	-0,1886	0,1833	N/S
REG	HERV-H	IL6	0,1552	0,9525	N/S
YC	HERV-H	IL17	-0,1952	0,3373	N/S
INAC	HERV-H	IL17	-0,4623	0,0200	*
REG	HERV-H	IL17	0,0331	0,8371	N/S
YC	HERV-H	IL1β	-0,1239	0,5463	N/S
INAC	HERV-H	IL1β	-0,3764	0,0442	N/S
REG	HERV-H	IL1β	0,0417	0,4756	N/S
YC	HERV-H	IL10	-0,0657	0,3607	N/S
INAC	HERV-H	IL10	-0,0933	0,3303	N/S
REG	HERV-H	IL10	0,3516	0,8789	N/S
